# Erythrodermie desquamative

**DOI:** 10.11604/pamj.2017.27.18.12273

**Published:** 2017-05-08

**Authors:** Majda Askour, Christelle Ebongo, Hafsae Bounniyt, Karima Senouci, Badredine Hassam

**Affiliations:** 1 Service de Dermatologie et Vénérologie, Centre Hospitalier Universitaire Ibn Sina, Faculté de Médecine et de Pharmacie, Université Mohammed V, Rabat, Maroc

**Keywords:** Erythrodermie, myélome multiple, dermatoses paranéoplasiques, Erythrodermia, multiple myeloma, paraneoplastic dermatoses

## Abstract

Les dermatoses paranéoplasiques sont un ensemble de signes cutanés qui peuvent précéder, coïncider ou suivre le diagnostic du cancer. Notre observation rappelle que l’érythrodermie desquamative fait partie des dermatoses paranéoplasiques facultatives au cours des hémopathies, d’où l’importance de faire un bilan complet devant ce signe clinique à la recherche d’un processus néoplasique interne, surtout quand la clinique est suspecte.

## Abstract (en)

Paraneoplastic dermatoses are a spectrum of cutaneous manifestations which may precede, coincide with or follow the diagnosis of cancer. Our study aims to remind clinicians that desquamative erythrodermia is a form of paraneoplastic dermatosis which may occur during hematologic malignancies. Hence the importance of a complete assessment to identify a neoplastic process in the presence of these clinical signs and especially when they are suspect.

## Introduction

Les dermatoses paranéoplasiques doivent être bien connues du dermatologue mais aussi de tous les internistes en raison de leur grand intérêt diagnostique. Elles peuvent être un signe de découverte d’une néoplasie profonde non encore diagnostiquée permettant alors une prise en charge précoce. Ou encore, elles peuvent être un signe de rechute d’une néoplasie connue et traitée. Notre observation illustre l’importance de chaque signe clinique, surtout quand l’anamnèse est suspecte.

## Patient et observation

**Figure 1:**
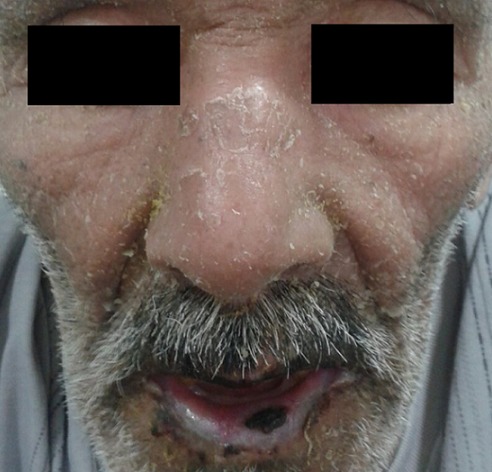
Erythrodermie desquamative avec une chéilite fissuraire

**Figure 2:**
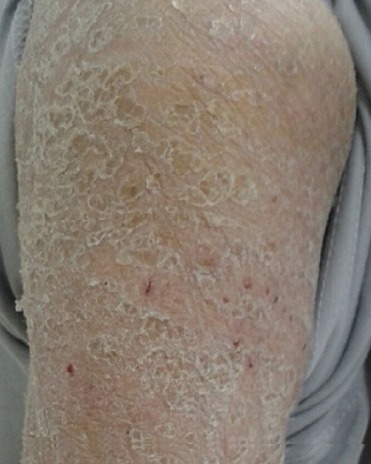
Desquamation cutanée diffuse avec des lésions excoriées par le grattage

Patient de 72 ans, avec antécédent de diabète type 1 sous régime, présente 10 jours avant son hospitalisation une érythrodermie prurigineuse avec œdème du visage et des membres inferieurs. L’évolution était marquée par la régression spontanée, avec desquamation cutanée diffuse. L’examen à l’admission notait la présence d’une érythrodermie desquamative, des lésions excoriées par le grattage et une chéilite fissuraire (Figure 1, Figure 2). Le reste de l’examen clinique était sans particularité. L’interrogatoire avait objectivé une prise d’amoxicilline protégée pour des angines pendant huit jours, trois semaines avant le début de sa symptomatologie cutanée. Le patient rapportait aussi la notion d’anorexie depuis un mois, et une altération de l’état général avec amaigrissement non chiffré. L’examen histologique de la biopsie cutanée était non spécifique. Les examens biologiques ont mis en évidence une anémie à 6,2 g/dl, normchrome normocytaire. Une vitesse de sédimentation à 140 mm à la première heure, avec une protéine C réactive de valeur normale. Le bilan hépatique et rénal était sans anomalies. On notait une hyponatrémie corrigée à 126 mEq/l, une hyperprotidémie à 115 g/l, avec une hypoalbuminémie à 19 g/L. L’électrophorèse des protéines sériques a mis en évidence un pic d’allure monoclonale en zone Gamma. Le complément par immunofixation a objectivé une immunoglobuline monoclonale d’isotype IgG Kappa, et la protéinurie de Bence jones était en faveur de chaine légère libre de type Kappa. Le bilan phosphocalcique était normal. Le diagnostic retenu était un myélome multiple, mais le patient a refusé de continuer les investigations, et il est sorti contre avis médical.

## Discussion

Les dermatoses paranéoplasiques (DPN) sont un ensemble de signes cutanés qui ne sont pas liées à un envahissement métastatique, mais elles sont considérées comme la conséquence indirecte de l’évolution d’un processus néoplasique interne. Leur particularité, est de précéder, coïncider ou suivre le diagnostic du cancer. On distingue deux types de syndromes paranéoplasiques cutanés, les DPN vraies ou spécifiques, et les DPN non spécifiques ou facultatives, dont l’érythrodermie desquamative [1, 2]. La particularité de notre observation, c’est que le patient avait déjà une notion de prise médicamenteuse quelques jours avant l’installation de la symptomatologie, faisant évoquer une allergie médicamenteuse. Mais l’âge du patient et l’interrogatoire qui a révélé un contexte général particulier, nous ont incité à faire un bilan général à la recherche d’une pathologie sous jacente, ce qui nous a permis de redresser le diagnostic vers une étiologie plus maligne, qui est le myélome multiple. L'érythrodermie est un érythème généralisé touchant 90 % de la surface cutanée, d’évolution prolongée, et associé à une desquamation diffuse. L'aspect clinique ne préjuge pas de son étiologie, qui peut être-selon le contexte clinique-une pathologie relativement bénigne, telles que le psoriasis et les allergies médicamenteuses, comme elle peut être maligne, ce qui était le cas de notre patient [3, 4].

## Conclusion

Notre observation rappelle que l’érythrodermie desquamative fait partie des dermatoses paranéoplasiques au cours des hémopathies.

## Conflits d’intérêts

Les auteurs ne déclarent aucun conflit d'intérêts.
